# Correction: Function of hTim8a in complex IV assembly in neuronal cells provides insight into pathomechanism underlying Mohr-Tranebjærg syndrome

**DOI:** 10.7554/eLife.56968

**Published:** 2020-03-18

**Authors:** Yilin Kang, Alexander J Anderson, Thomas Daniel Jackson, Catherine S Palmer, David P De Souza, Kenji M Fujihara, Tegan Stait, Ann E Frazier, Nicholas J Clemons, Deidreia Tull, David R Thorburn, Malcolm J McConville, Michael T Ryan, David A Stroud, Diana Stojanovski

Kang Y, Anderson AJ, Jackson TD, Palmer CS, De Souza DP, Fujihara KM, Stait T, Frazier AE, Clemons NJ, Tull D, Thorburn DR, McConville MJ, Ryan MT, Stroud DA, Stojanovski D. 2019. Function of hTim8a in complex IV assembly in neuronal cells provides insight into pathomechanism underlying Mohr-Tranebjærg syndrome. *eLife* **8**:e48828. doi: 10.7554/eLife.48828.

Published 4, November 2019

We have identified two typographical errors in the Material and Methods section of the manuscript. These errors were unintentional and have been rectified. See below.

[1] Under the heading - 'Cell lines and culturing, siRNA transfection, transient protein expression and stable cell line generation':

“For enhanced respiratory capacity cells were cultured in DMEM supplemented with 10 mM galactose, 10% dialyzed FBS, 1 mM sodium pyruvate, 50 mg/ mL uridine, and 0.01% penicillin/streptomycin.”

The concentration of Uridine should read 50 **μg**/ml NOT 50 mg/mL.

[2] Under the heading - 'In vitro protein import and autoradiography':

“Radiolabeled proteins were incubated with isolated mitochondria resuspended at 1 mg/ml in import buffer (20 mM HEPES-KOH (pH 7.4), 250 mM sucrose, 5 mM magnesium acetate and 80 mM magnesium acetate) supplemented with 10 mM sodium succinate, 1 mM 1,4-dithiothreitol and 5 mM ATP.”

This should read 5 mM magnesium acetate and 80 mM **potassium** acetate.

The article has been corrected accordingly.

